# Bmal1 haploinsufficiency impairs fear memory and modulates neuroinflammation via the 5-HT2C receptor

**DOI:** 10.3389/fphar.2024.1422693

**Published:** 2024-11-14

**Authors:** Weifen Li, Shengnan Mou, Tahir Ali, Tianxiang Li, Yan Liu, Shupeng Li, Xiaoming Yu, Zhi-Jian Yu

**Affiliations:** ^1^ School of Pharmacy, Shenzhen University Medical School, Shenzhen University, Shenzhen, China; ^2^ Department of Infectious Diseases and Shenzhen Key Laboratory for Endogenous Infections, The 6th Affiliated Hospital of Shenzhen University Health Science Center, Shenzhen, China; ^3^ State Key Laboratory of Oncogenomics, School of Chemical Biology and Biotechnology, Peking University Shenzhen Graduate School, Shenzhen, China; ^4^ Shenzhen Bay Laboratory, Shenzhen, China; ^5^ The Seventh Affiliated Hospital of Sun Yat-Sen University, Shenzhen, China; ^6^ Department of Psychiatry, University of Toronto, Toronto, ON, Canada; ^7^ Cancer Center, The Second Hospital, Cheeloo College of Medicine, Shandong University, Jinan, China

**Keywords:** Bmal1, learning and memory defects, 5-HT2CR, neuroinflammation, neurotransmitter

## Abstract

**Background:**

BMAL1, a key regulator of circadian rhythms, plays a multifaceted role in brain function. However, the complex interplay between BMAL1, memory, neuroinflammation, and neurotransmitter regulation remains poorly understood. To investigate these interactions, we conducted a study using BMAL1-haplodeficient mice (BMAL1^+/−^).

**Methods:**

We exposed BMAL1^+/−^ mice to behavioral assessments including cued fear conditioning, new objection recognition (NOR) test, and Y-maze test to evaluate BMAL1^+/−^ haplodeficiency impact on memory. Furthermore, biochemical changes were analyzed through western blotting, and ELISA to explore further the mechanism of BMAL1^+/−^ in memory, and neuroinflammation.

**Results:**

We found that BMAL1 haploinsufficiency led to deficits in cued fear learning and memory, while spatial memory and object recognition remained intact. Further analysis revealed dysregulated neurotransmitter levels and alterations in neurotransmitter-related proteins in the prefrontal cortex of BMAL1^+/−^ mice. Pharmacological interventions targeting dopamine uptake or the 5-HT2C receptor demonstrated that inhibiting the 5-HT2C receptor could rescue fear learning and memory impairments in BMAL1^+/−^ mice. Additionally, we observed downregulation of the inflammasome and neuroinflammation pathways in BMAL1^+/−^ mice, which is validated by inflammation mediator lipopolysaccharide (LPS) administration.

**Conclusion:**

These findings highlight that BMAL1 haploinsufficiency leads to deficits in fear learning and memory, which are linked to alterations in neurotransmitters and receptors, particularly the 5-HT2C receptor. Targeting the 5-HT2C receptor may offer a potential therapeutic strategy for mitigating cognitive impairments associated with BMAL1 dysfunction.

## 1 Introduction

Brain and muscle ARNT-Like 1 (Bmal1) is a crucial regulator of circadian rhythms but also plays significant roles in neuroinflammation, neuronal development, and dopamine signaling. These functions contribute to psychiatric disorders, neurodegenerative pathologies, memory retrieval, and glial cell (astrocyte) function (Hogenesch et al., 1998; Zheng et al., 2023). Previous studies have demonstrated Bmal1’s involvement in inflammatory and intracellular immune responses, as evidenced by the increased reactive oxygen species accumulation, upregulation of hypoxia-responsive protein (HIF-1α), and subsequent stimulation of pro-inflammatory cytokine IL-1β production observed in Bmal1-knockout (KO) macrophages (Tannahill et al., 2013; Early et al., 2018; Timmons et al., 2021; Zheng et al., 2023). Furthermore, global Bmal1 KO exhibited a range of neuropathological changes, including astrogliosis, synaptic degeneration, and impaired neuronal functional connectivity in the cortex and hippocampus (Musiek et al., 2013; Zheng et al., 2023). These findings underscore the importance of Bmal1 in maintaining brain health and function.

Learning and memory are essential for survival and adaptation (Brem et al., 2013; Guo et al., 2023). Bmal1 plays a crucial role in various learning and memory tasks, including spatial working memory and contextual fear memory (Price et al., 2016). Bmal1 can indirectly influence memory by regulating the neurotransmitter levels and signaling pathways. For instance, Bmal1 may affect dopaminergic elements, leading to altered neurotransmitter receptor expression and sensitivity (Kiehn et al., 2023). Additionally, Bmal1 affects serotonin signaling via circadian rhythms, subsequently influencing mood and impacting depression pathogenesis (Mieda and Sakurai, 2011).

Neuroinflammation is linked to memory defects and can be influenced by neurotransmitters (Jacqueline et al., 2017). Studies have shown that neuroinflammation can impair cognitive functions, particularly episodic memory. For example, individuals with higher levels of neuroinflammatory markers exhibit poorer episodic memory performance (Jacqueline et al., 2017; Dong et al., 2020; Wu and Zhang, 2023). Additionally, neuroinflammation contributes to memory deficits by dysregulating synaptic connectivity and potentially involving 5-HT receptors (Mottahedin et al., 2017). On the other hand, Bmal1 is a key regulator of neuroinflammation, influencing the expression of inflammatory genes and modulating microglial activity. Bmal1 deficiency has been linked to increased basal IL-6 levels in hypothalamic neurons and exacerbated microglial-mediated neuroinflammation in Parkinson’s disease models (Liu W. W. et al., 2020; Tran et al., 2020). Additionally, Bmal1 regulates microglial immune responses by inhibiting pro-inflammatory cytokines and enhancing anti-oxidative factors (Liu W. W. et al., 2020; Wang et al., 2021). Despite the established roles of Bmal1 in memory, neuroinflammation, and neurotransmitter regulation, the complex interplay between Bmal1, memory, neuroinflammation, and 5-HT receptor regulation remains poorly understood.

To explore the underlying mechanisms linking Bmal1, memory, and neuroinflammation, we conducted a study using Bmal1-haplodeficient mice while choosing the PFC because the brain circuits involved in fear conditioning prominently feature the medial prefrontal cortex (mPFC), which plays a critical role in regulating fear responses. We found that Bmal1 haploinsufficiency was associated with deficits in cued fear learning and memory, dysregulations in neurotransmitter systems, and neuroinflammatory responses. Treatment with a dopamine uptake inhibitor or a 5-HT2C receptor antagonist restored freezing levels and upregulated NLRP3 and JAK2/STAT3 pathways in the prefrontal cortex of Bmal1^+/−^ mice after cued fear conditioning. These findings suggest that Bmal1 plays a critical role in memory and cognition and that its dysfunction can lead to neuroinflammatory responses and cognitive deficits.

## 2 Materials and methods

### 2.1 Animals

In this study, age-matched (8–10 weeks old) wild-type (Bmal1^+/+^) and Bmal1 heterozygote-knockout (Bmal1^+/−^) littermate mice were used for all experiments. All these mice were maintained on a C57BL/6J genetic background. Bmal1^+/−^ mice were obtained from Prof. Yan Liu (Sun Yat-sen University). Animals had *ad libitum* access to regular chow and water and were maintained at 22°C on a standard 12-h light: 12-h dark (LD) cycle. Bmal1^+/+^ and Bmal1^+/−^ alleles were confirmed by PCR genotyping, and the protein expression of Bmal1 was confirmed by Western blot (Supplementary Figure S1). The Institutional Animal Care and Use Committee of Peking University Shenzhen Graduate School approved all the animal experiments.

### 2.2 Drug administration and schedule

The present investigation comprises the following experiments

**FIGURE 1:**
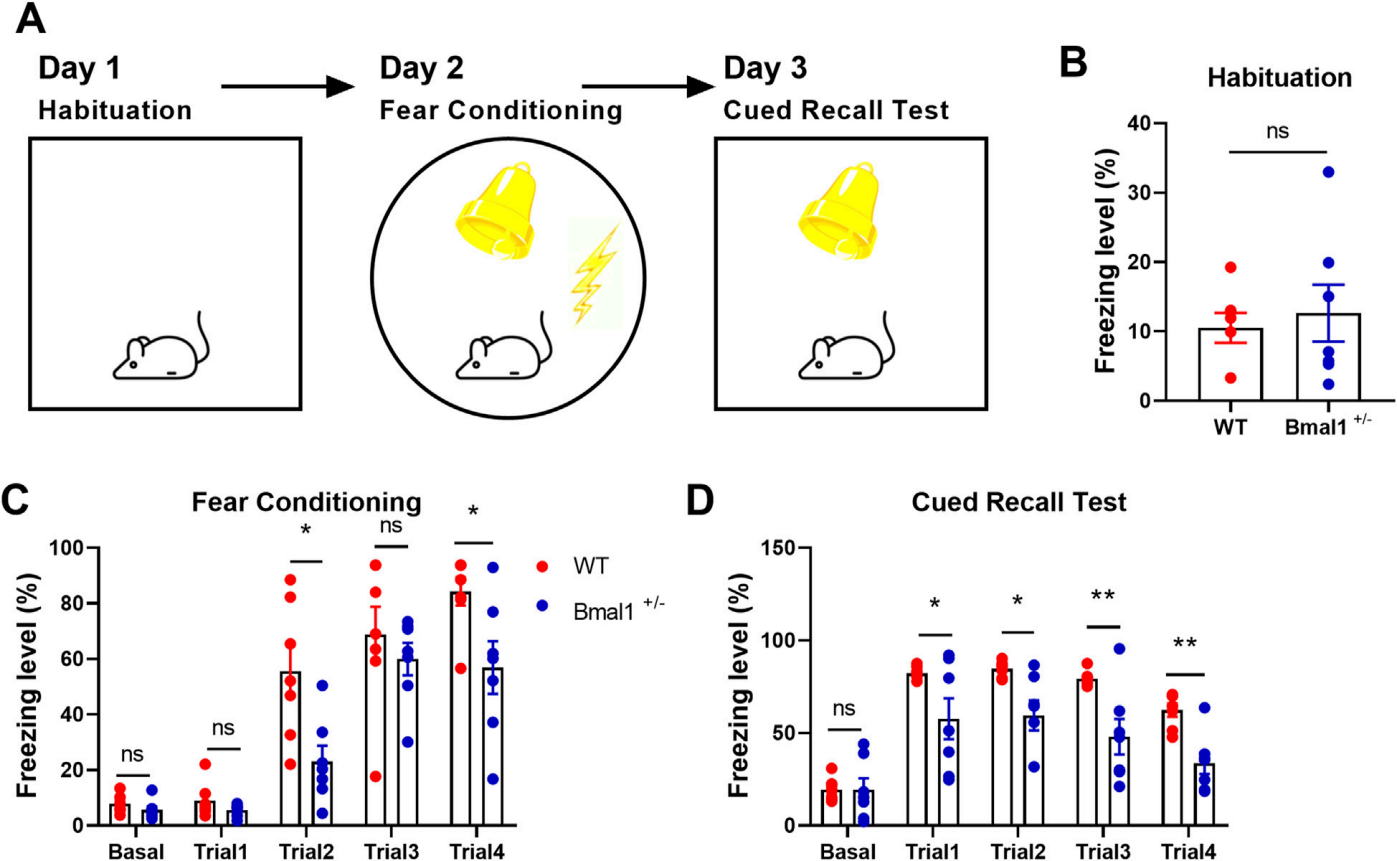
Fear learning and memory are impaired in Bmal1^+/−^ mice. **(A)** Schematic representation of the experimental timeline. In brief, mice were subjected to habitation, cued fear conditioning, cued recall test, and extinction training for four consecutive days. **(B–D)** Freezing levels of Bmal1^+/+^ and Bmal1^+/−^ mice in distinct tests. Bmal1^+/+^ mice, n = 7; Bmal1^+/−^ mice, n = 7. Data are reported as mean ± SEM. Unpaired t-test. ns, no significance; *p < 0.05; **p < 0.01.

Experiment 1: to investigate the functional role of Bmal1 in cued fear memory, the experimental male mice (wild-type mice, WT, n = 7; Bmal1 haploinsufficiency mice, Bmal1^+/−^, n = 7) performed habituation on day 1, followed by fear conditioning on day 2, and then a cued recall test on day 3 (Figure 1A). Habituation and cued recall test are the same context, fear conditioning is a different context, and the test method was followed by previous research (Yan et al., 2019).
Experiment 2: to further confirm the functional role of 5-HT and dopamine in cued fear learning and memory, vanoxerine dihydrochloride (VD, a selective dopamine uptake inhibitor, HY-13217, MedChemExpress, NJ, United States, 20 mg/kg dissolved in saline) or SB242084 (selective 5-HT2C receptor antagonist, HY-13409, MedChemExpress, NJ, United States, 1 mg/kg dissolved in saline) was, respectively, injected intraperitoneally 20 min before cued fear conditioning. The Bmal1^+/−^ male mice (saline + Bmal1+/− group, n = 7 mice; VD + Bmal1+/− group, n = 7 mice; SB242084 + Bmal1+/− group, n = 7 mice) underwent fear conditioning on day 1 and cued recall test on day 2. Mice were sacrificed 1.5 h after the cued recall test (Figure 3A).
Experiment 3: to explore whether Bmal1 is regulated by inflammation, adult male WT mice (saline group, n = 4; LPS group, n = 4) were injected intraperitoneally with 0.5 mg/kg lipopolysaccharide (LPS, L8880, Solarbio, Beijing, CHN) once daily for 7 consecutive days. Mice were sacrificed for Bmal1 protein assays (Figure 6A).
Experiment 4: to further confirm whether LPS can improve fear learning dysfunction in the Bmal1 haploinsufficiency condition, Bmal1^+/−^ mice (Bmal1^+/−^ group, n = 6; Bmal1^+/−^ + LPS group, n = 6) were injected intraperitoneally with 0.5 mg/kg lipopolysaccharide once daily for 7 consecutive days. Mice underwent fear conditioning on day 1 and cued recall test on day 2. Mice were sacrificed after 1.5 h of the cued recall test (Figure 6B).

### 2.3 Cued fear conditioning

The FreezeFrame system (Coulbourn Instruments) was used for fear conditioning during all the experiments. Training was performed in an experiment chamber that consisted of black methacrylate walls and a transparent front door. On the training day, each mouse was placed in the chamber and allowed to explore it for 2 min, after which the mouse received four pairings of the 30-s, 2.5-kHz auditory cue (80 dB tone, cage enclosed) co-terminating with a 2-s, 0.8-mA footshock. The inter-trial interval was 60 s. Following the last footshock, the mouse was allowed to explore the chamber for an additional 1 min before being returned to its home cage.

The recall test was performed in a cage with a different context (scent: 1% acetic acid) from the training cage used in fear conditioning. The cage was equipped with non-shocking grids. Mice were habituated for 2 min and received five presentations of the same auditory tone used in fear conditioning. The inter-trial interval was 60 s. One minute after the test, the mice were returned to their home cages.

Cued fear conditioning and recall tests were conducted during the day.

### 2.4 Behavioral assays

#### 2.4.1 New objection recognition (NOR) test

The new objection recognition (NOR) was performed according to our previous report (Liu et al., 2023). Mice were placed into an open-field apparatus (45 × 45 × 30 cm) with a video camera mounted on the chamber. In brief, mice were placed into the training box and were allowed to freely explore two identical objects for 10 min on day 1 (training session). On day 2 (reactivation session), we placed the mice into the training box and allowed them to explore the same two identical objects for 5 min freely. On day 3 (test session), the mice were re-introduced into the training box, in which one of the objects was randomly replaced by a novel object and were allowed to explore the two objects for 5 min freely. All objects presented similar textures and sizes but had distinctive shapes and colors. Exploration was defined as sniffing the object with the nose at a distance of no more than 2 cm and/or touching it with the nose or forepaws. Sitting on the object was not considered exploration. The field and objects were thoroughly wiped down using 75% ethanol between each testing trial. The time spent exploring each object and the total time spent exploring both objects were recorded manually for all test periods. The recognition object index was calculated using the following formula: index = TN/(TN + TF) (where TN is the time spent exploring the novel object and TF is the time spent exploring the familiar object in the test session).

### 2.5 Y-maze test

The Y-maze test was used to evaluate the spatial working memory of the mice. Each maze consisted of three equal arms (30 cm length × 5 cm width × 15 cm height) termed novel, start, and other, which were at a 120° angle from each other. The ends of each arm were marked with papers of different shapes and colors. Mice were placed in the center and allowed to explore the maze for 8 min freely. The ANY-maze software was used to record the time and entries into the three arms and the distance moved. The percentage of alteration was calculated as the ratio of actual to possible alterations (defined as the total number of arm entries – 2) × 100. After each test, mice were immediately returned to their home cages, and the arms were cleaned with 75% ethanol to avoid any possible instinctive odorant cues (Liu et al., 2023).

### 2.6 Enzyme-linked immunosorbent assay (ELISA)

The expression levels of IL-6 (ABclonal, cat no: RK00008), TNF-α (ABclonal, cat no: RK00027), IL-1β (ABclonal, cat no: RK00006), IL-10 (ABclonal, cat no: RK00016), IL-4 (ABclonal, cat no: RK00036), TGFβ (ABclonal, cat no: RK00057), CORT (Mlbio, ml037564), 5-HT (ABclonal, cat no: RK09044), DA (ABclonal, cat no: RK00642), Ach (Mlbio, ml401805), GABA (Mlbio, ml092740), GLU (ABclonal, cat no: RK04527), and NE (Mlbio, ml063805) in the prefrontal cortex (PFC) were measured using commercial ELISA kits, according to the manufacturer’s instructions (Li W. et al., 2023).

### 2.7 Western blot

Tissue samples from the PFC were homogenized in ice-cold lysis buffer and then centrifuged at 3,000 g for 10 min at 4°C to extract the supernatants. The protein concentrations were quantified using the BCA assay. All the normalized samples were separated using 10% SDS-PAGE gels and then transferred onto polyvinylidene difluoride (PVDF) membranes. The membranes were blocked with 5% bovine serum albumin for 1 h at room temperature and then incubated with the following primary antibodies shown in Table 1. The membranes were washed with TBST and then incubated with secondary antibodies (rabbit or mouse antibody; 1:5,000) for 1 h at room temperature. ImageJ software was used to calculate the gray value of immune reactivity. The representative Western blots of the target proteins were cropped and are presented along with their corresponding control GAPDH in the figures in the main text (Li W. et al., 2023).

**TABLE 1:** Detailed antibody information.

Antibody name	Company	Lot number	Dilute
5-HT2CR	Santa Cruz	SC-166775	1:500
IDO	Cell Signaling Technology	#51851	1:1000
TPH2	Cell Signaling Technology	#51124	1:1000
TH	Cell Signaling Technology	#2792	1:1000
DAT	Abcam	ab128848	1:1000
p-TrkB	Abcam	ab229908	1:1000
TrkB	Cell Signaling Technology	#4603	1:1000
PSD95	Abcam	ab238135	1:1000
SNAP25	Cell Signaling Technology	#5308	1:1000
Synapsin-1	Cell Signaling Technology	#25297	1:1000
Synaptophysin	Cell Signaling Technology	#36406	1:1000
p-IP3	Cell Signaling Technology	#12243	1:1000
IP3	Cell Signaling Technology	#8568	1:1000
p-PKC	Cell Signaling Technology	#9379	1:1000
PKC	Cell Signaling Technology	#2056	1:1000
p-CREB	Cell Signaling Technology	#9198	1:1000
CREB	Cell Signaling Technology	#9197	1:1000
SIRT1	Cell Signaling Technology	#8469	1:1000
p-MEK	Cell Signaling Technology	#9121	1:1000
MEK	Cell Signaling Technology	#9122	1:1000
p-ERK	Cell Signaling Technology	#9101	1:1000
ERK	Proteintech	16443-1-AP	1:1000
p-JNK	Cell Signaling Technology	#4668	1:1000
JNK	Cell Signaling Technology	#9252	1:1000
p-P38	Cell Signaling Technology	#4511	1:1000
P38	Cell Signaling Technology	#9212	1:1000
p-eEF2	Cell Signaling Technology	#2331	1:1000
eEF2	Abcam	ab33523	1:1000
p-eIF2α	Cell Signaling Technology	#3597	1:1000
eIF2α	Cell Signaling Technology	#9722	1:1000
p-eIF4E	Cell Signaling Technology	#9741	1:1000
eIF4E	Cell Signaling Technology	#2067	1:1000
p-JAK1	Cell Signaling Technology	#74129	1:1000
JAK1	Cell Signaling Technology	#3344	1:1000
p-JAK2	Cell Signaling Technology	#3771	1:1000
JAK2	Cell Signaling Technology	#3230	1:1000
p-JAK3	Cell Signaling Technology	#5031	1:1000
JAK3	Cell Signaling Technology	#8863	1:1000
p-STAT1(Ser727)	Cell Signaling Technology	#8826	1:1000
STAT1	Proteintech	10144-2-AP	1:1000
p-STAT3(Ser705)	Cell Signaling Technology	#9145	1:1000
STAT3	Cell Signaling Technology	#9139	1:1000
p-AMPKα	Cell Signaling Technology	#2535	1:1000
AMPKα	Cell Signaling Technology	#5832	1:1000
Complex Ⅰ	ABclonal	A10123	1:2000
Complex Ⅱ	Abcam	ab14714	1:200
Complex Ⅲ	Abcam	ab14746	1:1000
Complex Ⅳ	Abcam	ab110262	1:1000
Complex Ⅴ	Abcam	ab14748	1:1000
PGC-1α	Cell Signaling Technology	ab54481	1:1000
TFAM	Proteintech	22586-1-AP	1:1000
DRP1	Cell Signaling Technology	#8570S	1:1000
OPA1	Cell Signaling Technology	#80471S	1:1000
MFN1	Santa Cruz	SC-166644	1:500
NLRP3	Cell Signaling Technology	#15101	1:1000
NFκB	Cell Signaling Technology	#8242	1:1000
p-IKK	Cell Signaling Technology	#2694S	1:1000
IKK	Cell Signaling Technology	#2370S	1:1000
Bmal1	Abcam	ab235577	1:1000
p-PKA	Cell Signaling Technology	#9621	1:1000
PKA	Cell Signaling Technology	#3927	1:1000
GAPDH	Cell Signaling Technology	#5174	1:1000

### 2.8 Statistical analysis

Western blot band intensities were quantified using ImageJ (version 1.30) and analyzed with GraphPad Prism 8. Data are presented as mean ± standard error of the mean (SEM). Statistical significance was assessed using t-tests for pairwise comparisons or one-way ANOVA, followed by Tukey’s *post hoc* multiple comparisons test. A significance level of p < 0.05 was adopted for all statistical analyses.

## 3 Results

### 3.1 Bmal1^+/−^ haploinsufficiency induced fear learning and memory defects

Previous reports have shown that Bmal1 deficiency could induce contextual fear memory and spatial memory impairment (Wardlaw et al., 2014; Price and Obrietan, 2018). In the present study, we investigated the functional role of Bmal1 in cued fear memory. Bmal1^+/+^ and Bmal1^+/−^ mice were first habituated in context A and then underwent cued fear conditioning in a novel context (context B), followed by a recall test in context A and extinction training in context B. Bmal1^+/−^ mice showed the attenuated freezing level during cued fear conditioning (trail 2: *p* = 0.0188, F 2.235, trail 4: *p* = 0.0250, F 3.353) and the subsequent recall test (Figures 1A–D) (trail 1: *p* = 0.049, F 61.42, trail 2: *p* = 0.0104, F 26.27, trail 3: *p* = 0.0076, F 35.22, trail 4: *p* = 0.0013, F 2.686). However, Bmal1^+/−^ did not affect the total moved distance and the exploration of the new route compared to the old route (Supplementary Figure S2B, C). In addition, the preference index was comparable between Bmal1^+/+^ and Bmal1^−/−^ mice (Supplementary Figure S2A). These results demonstrate that Bmal1^+/−^ mice exhibit abnormal conditioned fear learning and memory, normal spatial memory, and normal object recognition ability.

### 3.2 Bmal1^+/−^ haploinsufficiency impaired neurotransmitter and receptors, including 5-HT2CR

**FIGURE 2:**
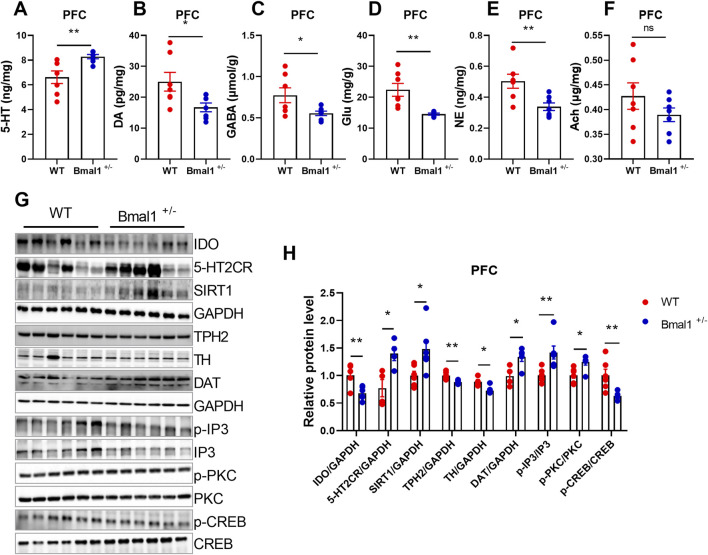
Bmal1^+/−^ mice show abnormal neurotransmitter levels and molecular signals. **(A–F)** Levels of neurotransmitters in the PFC of Bmal1^+/+^ and Bmal1^+/−^ mice after cued fear conditioning paradigm. 5-HT **(A)**, dopamine **(B)**, GABA **(C)**, glutamate **(D)**, norepinephrine **(E)**, and acetylcholine **(F)**. n = 7 mice. Data are reported as mean ± SEM. Unpaired t-test. ns, no significance; *p < 0.05; **p < 0.01. **(G, H)** Representative Western blot images **(G)** and protein levels of IDO, 5-HT2CR, SIRT1, TPH2, TH, DAT, p-IP3, p-PKC, and p-CREB in the PFC of Bmal1^+/+^ and Bmal1^+/−^ mice after the cued fear conditioning paradigm **(H)**. n = 4–6 mice. Data are reported as mean ± SEM. Unpaired t-test. ns, no significance; *p < 0.05; **p < 0.01.

Studies have demonstrated that Bmal1 depletion disrupts circadian gene expression in the brain nuclei, particularly those involved in neurotransmitter-related functions in the ventral striatum, suggesting that Bmal1 dysfunction may impact these behaviors through its influence on neurotransmitter signaling (Hasegawa et al., 2019). To elucidate the molecular mechanisms, we analyzed neurotransmitter levels and related gene expression. We found increased levels of 5-HT levels (p = 0.0092, F, 8.499), while dopamine (DA) (p = 0.0292, F, 4.552), GABA (p = 0.0383, F, 11.11), glutamate (*p* = 0.0029, F, 42.03), and norepinephrine (NE) (*p* = 0.0077, F, 3.372) in Bmal1^+/−^ mice were decreased (Figures 2A–F) in the Bmal1^+/−^ prefrontal cortex (PFC). Additionally, the expression of SIRT1 (*p* = 0.0277, F, 5.051), 5-HT2C receptor (*p* = 0.0204, F, 1.545), and DAT (*p* = 0.0226, F, 1.006) was upregulated, while IDO (*p* = 0.0051, F, 2.563), TPH2 (*p* = 0.0023, F, 4.698), and TH (*p* = 0.0137, F, 1.386) were downregulated in Bmal1^+/−^ mice (Figures 2G, H). We further examined downstream molecules in the 5-HT pathway and found altered ratios of p-IP3/total IP3 (*p* = 0.0089, F, 5.522), p-PKC/total PKC (*p* = 0.0118, F, 1.045), and p-CREB/total CREB (*p* = 0.0082, F, 16.84) in Bmal1^+/−^ mice (Figures 2G, H). These findings suggest that dysregulation of the neurotransmitter system, particularly within the 5-HT pathway, may contribute to the impaired cued fear learning and memory observed in Bmal1^+/−^ mice.

### 3.3 5-HT2C receptor inhibition rescues cued fear memory deficits in Bmal1-deficient mice

**FIGURE 3:**
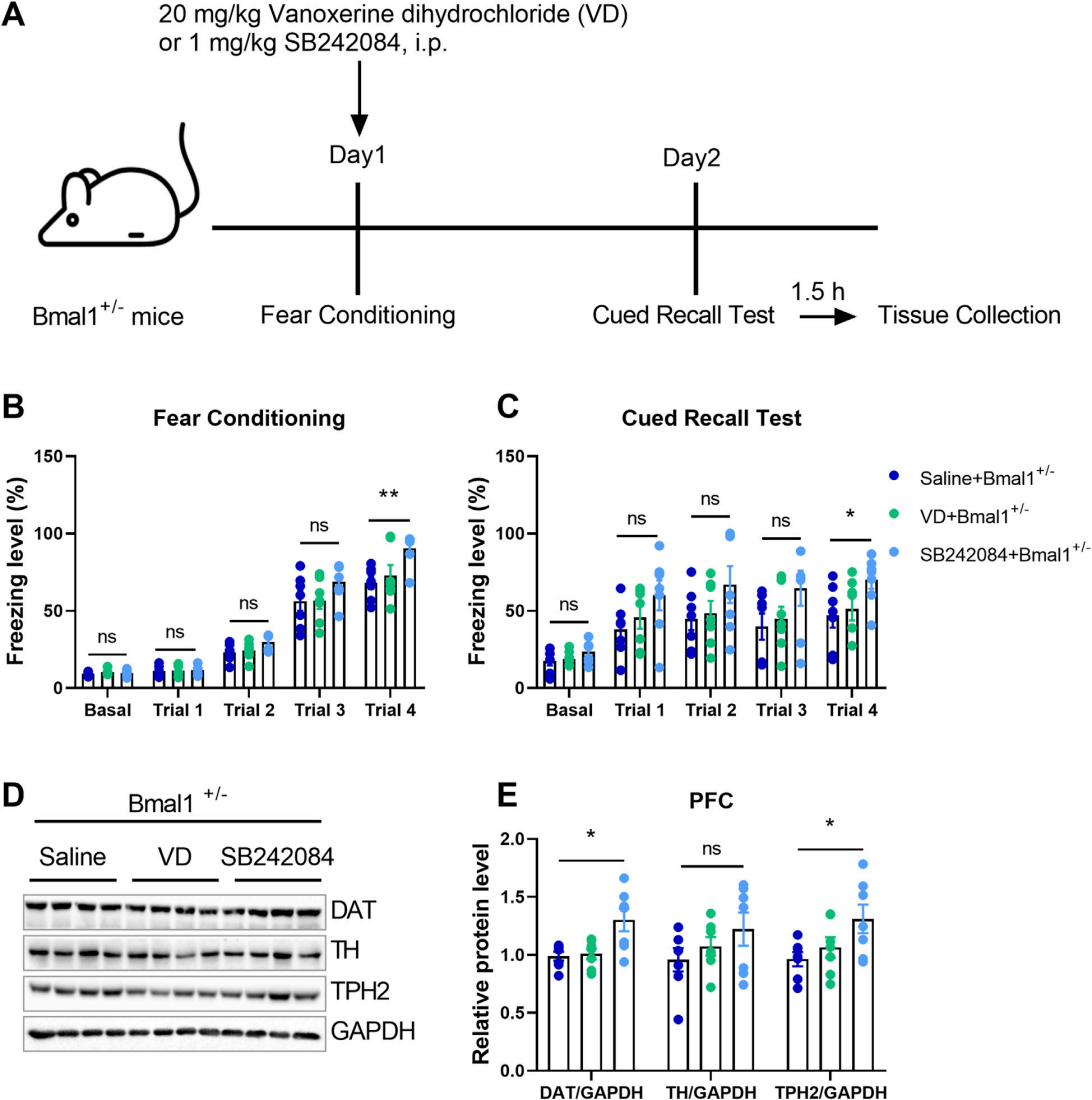
Pharmacological inhibition of the 5-HT2C receptor reversed the impairment of cued fear learning and memory in Bmal1^+/−^ mice. **(A)** Schematic representation of the experimental timeline. Vanoxerine dihydrochloride and SB242084 were administrated 20 min before cued fear conditioning. **(B, C)** Freezing levels of Bmal1^+/−^ mice in cued fear conditioning and the recall test. Saline + Bmal1^+/−^ group, n = 7 mice; VD + Bmal1^+/−^ group, n = 7 mice; SB242084 + Bmal1^+/−^group, n = 7 mice. Data are reported as mean ± SEM. Unpaired t-test. Saline + Bmal1^+/−^ group vs. VD + Bmal1^+/−^ group; saline + Bmal1^+/−^ group vs. SB242084+ Bmal1^+/−^ group. ns, no significance; *p < 0.05; **p < 0.01. **(D, E)** Representative Western blot images **(D)** and protein levels of DAT, TH, and TPH2 in the PFC of Bmal1^+/−^ mice after the cued fear conditioning paradigm **(E)**. n = 7 mice. Data are reported as mean ± SEM. Unpaired t-test. Saline + Bmal1^+/−^ group vs. VD + Bmal1^+/−^ group; saline + Bmal1^+/−^group vs. SB242084 + Bmal1^+/−^group. ns, no significance; *p < 0.05.

To elucidate the functional roles of 5-HT and dopamine in cued fear learning and memory, we administered vanoxerine dihydrochloride (VD), a selective dopamine uptake inhibitor, or SB242084, a selective 5-HT2C receptor antagonist, to Bmal1^+/−^ mice. As shown in Figure 3A, SB242084, but not VD, blocked the effects of Bmal1 haploinsufficiency on cued fear learning and memory (Figures 3B, C) (*p* = 0.028, 0.0412). Additionally, the SB242084 treatment restored DAT (*p* = 0.0100) and TPH2 (*p* = 0.045) expression levels (Figures 3D, E). These findings suggest that inhibiting the 5-HT2C receptor, but not dopamine uptake, can reverse the impairments in cued fear learning and memory observed in Bmal1^+/−^ mice.

### 3.4 The inflammasome and neuroinflammation pathways participate in the action of 5-HT in Bmal1^+/−^ mice

**FIGURE 4:**
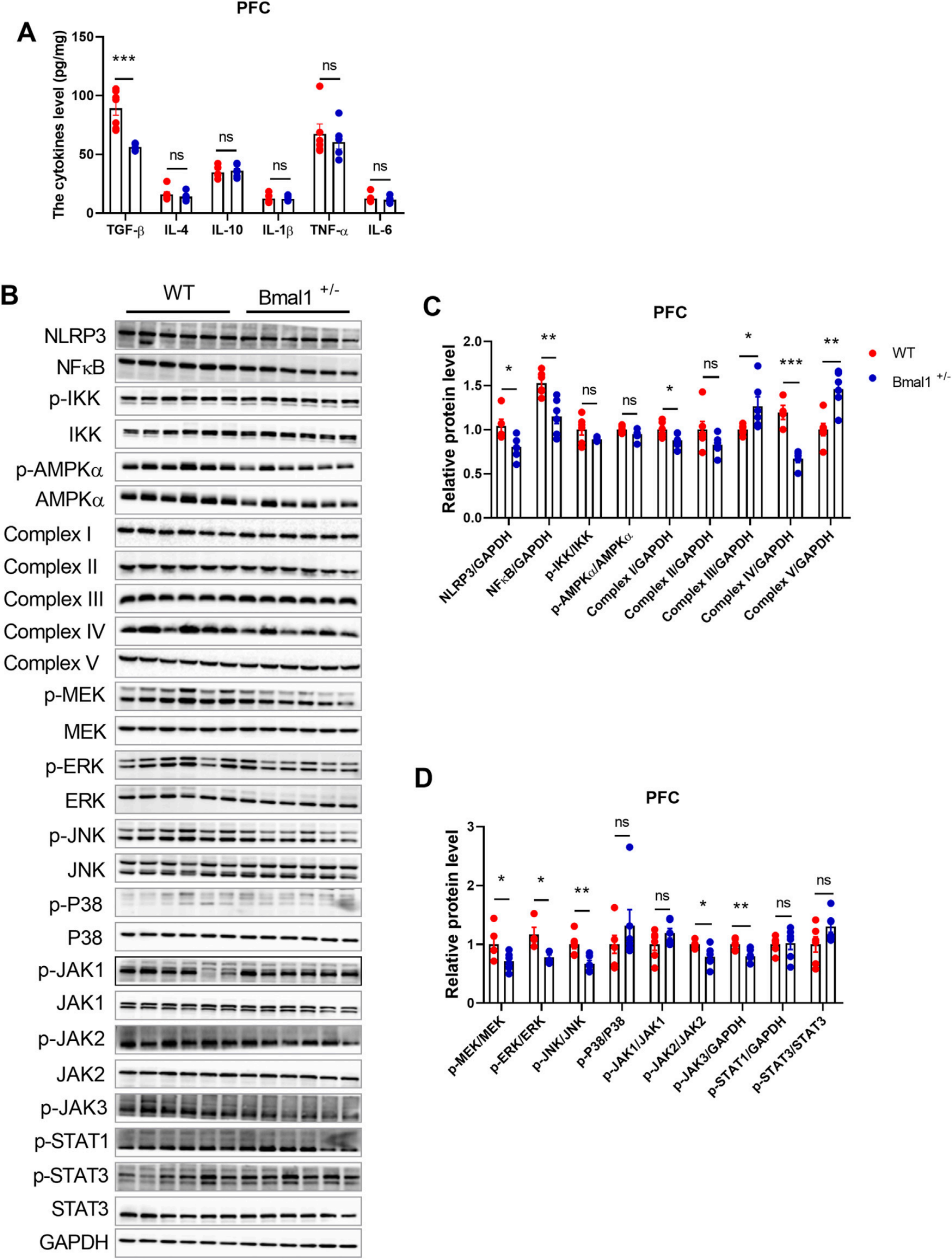
Bmal1^+/−^ modified the protein expression of inflammation, mitochondrion, MAPK pathway, and JAK-STAT pathway and decreased TGF-β in PFC. **(A)** ELISA of inflammatory cytokines measured in the PFC of Bmal1^+/+^ and Bmal1^+/−^ mice after the cued fear conditioning paradigm. **(B–D)** Representative Western blot images **(B)** and protein levels of NLRP3, NFκB, p-IKK, p-AMPKα, complex I–V, p-MEK, p-ERK, p-JNK, p-P38, p-JAK1, p-JAK2, p-JAK3, p-STAT1, and p-STAT3 in the PFC of Bmal1^+/+^ and Bmal1^+/−^ mice after the cued fear conditioning paradigm **(C, D)**. n = 4–6 mice. Data are reported as mean ± SEM. Unpaired t-test. ns, no significance; *p < 0.05; **p < 0.01; ***p < 0.001. n = 6–77 mice. Data are reported as mean ± SEM. Unpaired t-test. ns, no significance; *p < 0.05; **p < 0.01; ***p < 0.001.

Bmal1 has been implicated in inflammatory and intracellular immune responses, suggesting a potential crosstalk with the neurotransmitter system. To investigate the effects of Bmal1 deficiency on neuroinflammation, we measured cytokine levels and inflammatory-associated signaling in Bmal1^+/−^ mice PFC. Enzyme-linked immunosorbent assay (ELISA) revealed a significant downregulation of transforming growth factor beta (TGF-β) (*p* = 0.0001, F, 28.96) in Bmal1^+/−^ mice after cued fear memory conditioning. However, levels of IL-1β, IL-4, IL-6, IL-10, and TNF-α remained unchanged (Figure 4A). However, neuroinflammation-related signaling pathways, including MAPK/JNK, IKK/NF-κB, and JAK/STAT, were significantly influenced in Bmal1^+/−^ mice. The cued fear memory paradigm led to a decrease in the NLRP3 (*p* = 0.0285, F, 1.793) inflammasome and NF-κB (*p* = 0.0034, F, 3.373) in these mice. Furthermore, we found alterations in mitochondria-related pathways, including the upregulation of Complex III (*p* = 0.027, F, 1.195) and Complex V (*p* = 0.0019, F, 1.486) and the downregulation of Complex IV (*p* = 0.0005, F, 2.609) (Figures 4B, C) and PGC1-α (S3A, C), while no significant changes were observed in the expression AMPKα or p-AMPKα in the PFC of Bmal1^+/−^ mice. Additionally, MEK (*p* = 0.0384, F, 2.939), ERK (*p* = 0.0134, F, 6.692), and JNK (*p* = 0.0050, F, 1.877), as well as downregulated p-JAK2 (*p* = 0.0180, F, 8.582) and p-JAK3 (*p* = 0.0080, F, 1.604) phosphorylation, were decreased. However, we did not find changes in the p-STAT3 or STAT3 expression in the PFC tissue of the Bmal1^+/−^ mice (Figures 4B, D).

Studies have suggested that neuroinflammation could influence synaptic proteins (Jiang et al., 2022; Gong et al., 2023; Li A. et al., 2023). Hence, we examined changes in synaptic protein expression in the PFC of the Bmal1^+/−^ mice. The cued fear memory paradigm induced a decrease in PSD95 levels and an increase in synaptophysin levels in Bmal1^+/−^mice (Supplementary Figure S3A, B). Additionally, corticosterone levels were comparable between WT and Bmal1^+/−^ mice (Supplementary Figure S3D). These findings suggest that the downregulation of neuroinflammation-related signals contributes to the impairment of cued fear learning and memory in Bmal1^+/−^mice.

### 3.5 5-HT2C receptor inhibition rescues neuroinflammatory and mitochondrial dysfunction in Bmal1-deficient mice

**FIGURE 5:**
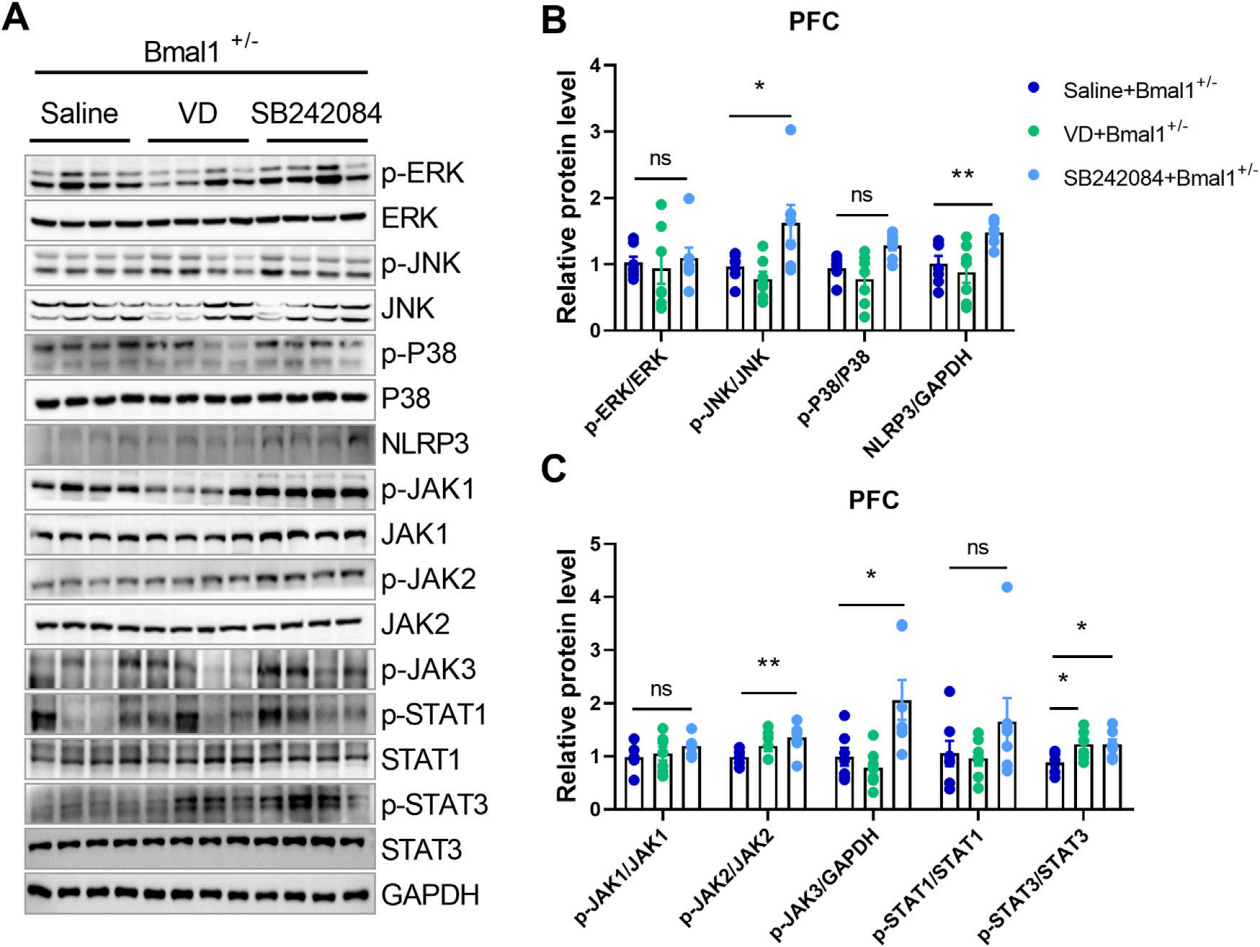
The NLRP3 inflammasome and neuroinflammation pathways participate in the action of 5-HT in Bmal1^+/−^ mice. **(A–C)** Representative Western blot images **(A)** and protein levels of the MAPK pathway and NLRP3 **(B)**, JAK/STAT pathway **(C)** in the PFC of Bmal1^+/−^ mice after the cued fear conditioning paradigm. n = 7 mice. Data are reported as mean ± SEM. Unpaired t-test. Saline + Bmal1^+/−^group vs. VD + Bmal1^+/−^ group; saline + Bmal1^+/−^ group vs. SB242084 + Bmal1^+/−^group. ns, no significance; *p < 0.05; **p < 0.01.

To investigate whether 5-HT2C or dopamine receptor inhibition could reverse neuroinflammatory changes in Bmal1^+/−^ mice, we treated them with VD or SB242084. Inhibition of the 5-HT2C receptor, but not VD, restored the downregulated MAPK/JNK and JAK/STAT pathways in the PFC of Bmal1^+/−^ mice (Figures 5A–C). The NLRP3 (*p* = 0.0335) inflammasome level in the prefrontal cortex (PFC) was also restored by SB242084 treatment, following the cued fear memory paradigm (Figures 5A, B). Furthermore, SB242084 treatment normalized p-JNK/total JNK, p-JAK2/total JAK2 (*p* = 0.0312), and p-JAK3/total JAK3 (*p* = 0.0398) ratios in the PFC (Figures 5A–C; Supplementary Figure S4). However, we did not observe changes in the expression levels of p-ERK/total ERK, p-P38/total P38, p-JAK1/total JAK1, p-STAT1/total STAT1, MFN1, TFAM, and Complex I–V (Figures 5A–C; Supplementary Figure S4). Interestingly, pharmacological inhibition of the 5-HT2C receptor significantly decreased the p-AMPK/total AMPK ratio in the PFC. In contrast, pharmacological inhibition of dopamine uptake primarily affected mitochondrial function, as evidenced by decreased PGC-1α and OPA1 expression levels (Supplementary Figure S4). These findings suggest that the impairments in cued fear learning and memory in Bmal1^+/−^ mice may primarily be mediated by alterations in the 5-HT2C receptor and the associated signaling pathway rather than mitochondrial dysfunction.

### 3.6 Inflammation mediates Bmal1-induced cued fear memory impairment

**FIGURE 6:**
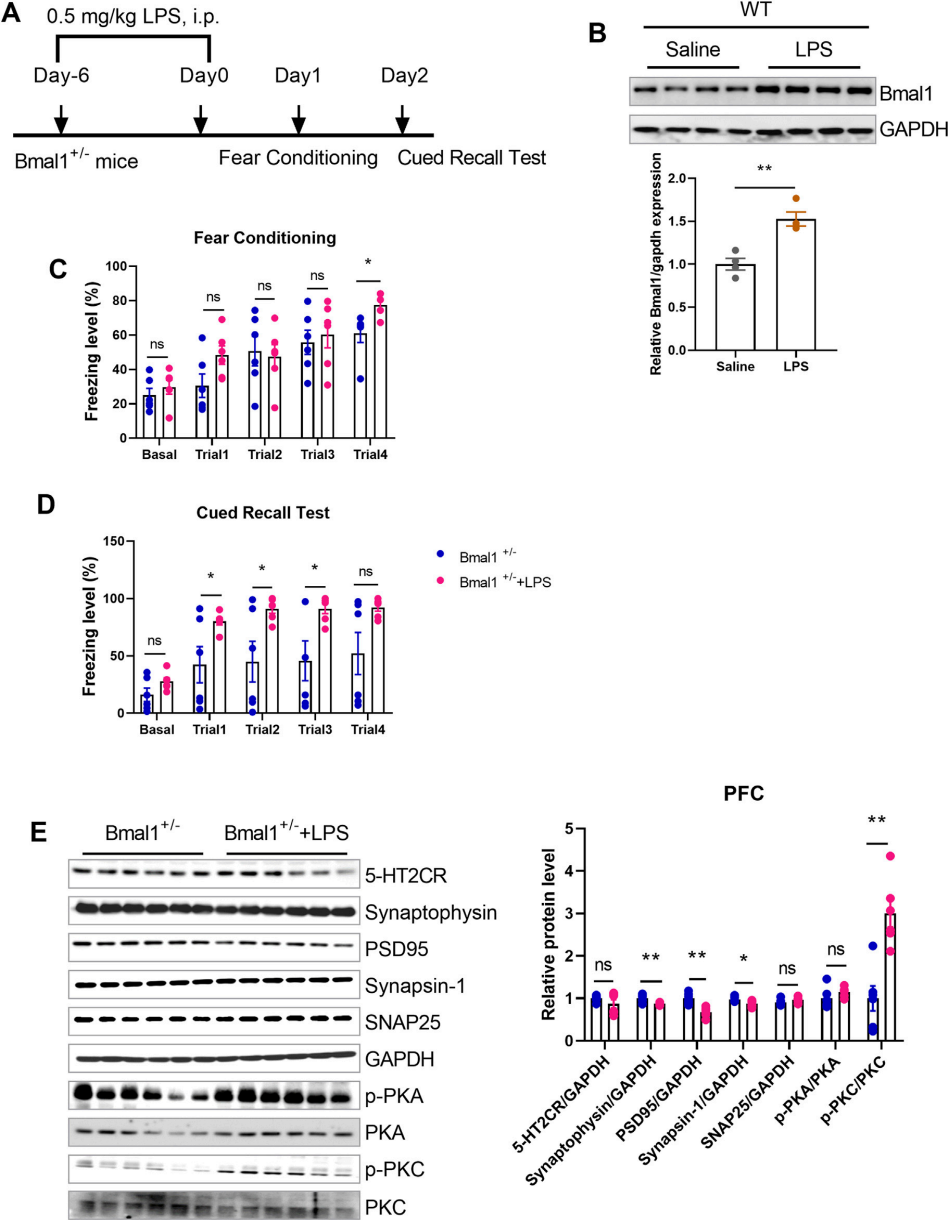
LPS reversed the impairment of cued fear learning and memory in Bmal1^+/−^ mice. **(A)** Representative Western blot images and protein levels of Bmal1 in the PFC of Bmal1^+/+^ and Bmal1^+/−^ mice after LPS injection. n = 6 mice. Data are reported as mean ± SEM. Unpaired t-test. ns, no significance; *p < 0.05; **p < 0.01. **(B)** Schematic representation of the experimental timeline. LPS was administrated 7 days before fear conditioning. n = 4 mice. **(C, D)** Freezing levels of Bmal1^+/−^ mice in cued fear conditioning and recall test. Bmal1^+/−^ group, n = 6 mice; Bmal1^+/−^ + LPS group, n = 6 mice. Data are reported as mean ± SEM. Unpaired t-test. Bmal1^+/−^ group vs. Bmal1^+/−^ + LPS group. ns, no significance; *p < 0.05; **p < 0.01. **(E)** Representative Western blot images and protein levels of 5-HT2CR, synaptophysin, PSD95, synapsin-1, SNAP25, p-PKA, and p-PKC in the PFC of Bmal1^+/−^ mice after LPS injection **(D)**. n = 6 mice. Data are reported as mean ± SEM. Unpaired t-test. Bmal1^+/−^group vs. Bmal1^+/−^ + LPS group. ns, no significance; *p < 0.05; **p < 0.01.

To investigate the role of inflammation in cued fear learning and memory in Bmal1^+/−^ mice, we administered lipopolysaccharide (LPS) (Figure 6A), an inflammation mediator, to the WT mice. LPS treatment upregulated Bmal1 protein levels (*p* < 0.0001, F, 1.571) (Figure 6B) in wild-type mice and reversed the impaired cued fear learning and memory in Bmal1^+/−^ mice (*p* = 0.0298, F, 20.55) (Figures 6C, D). Additionally, LPS treatment downregulated synaptic proteins (synaptophysin (*p* = 0.0073, F, 7.511), PSD95 (*p* = 0.0010, F, 1.276), and synapsin-1 (*p* = 0.0242, F, 1.392)) but upregulated p-PKC (*p* = 0.0010, F, 1.199) in Bmal1^+/−^ mice. However, the expression of 5-HT2CR, SNAP25, and p-PKA remained unchanged (Figure 6E). These findings suggest that inflammation plays a significant role in the cued fear learning and memory changes associated with Bmal1 haploinsufficiency.

## 4 Discussion

This study highlights a significant interplay between Bmal1, neuroinflammation, and memory impairment, mediated in part by neurotransmitters and receptors, particularly the 5-HT2C receptor. Bmal1 haploinsufficiency was associated with fear learning and memory deficits, which were linked to alterations in neurotransmitters and receptors, including the 5-HT2C receptor. Notably, 5-HT2C receptor inhibition rescued these deficits in Bmal1^+/−^ mice. Furthermore, Bmal1^+/−^ mice exhibited changes in TGF-β levels and inflammatory synaptic signaling, following cued fear memory conditioning. Interestingly, LPS treatment reversed the impaired cued fear learning and memory in Bmal1^+/−^ mice, suggesting a potential role of inflammation in these processes.

A plethora of reports highlight Bmal1’s significant role in the pathophysiology of various psychiatric disorders and neurodegenerative diseases, including depression and memory defects (Wardlaw et al., 2014; Liu W. W. et al., 2020; Wang et al., 2021; Zheng et al., 2023). Previous studies have demonstrated that Bmal1^+/−^ mice exhibit contextual fear memory and spatial memory impairments (Wardlaw et al., 2014; Zheng et al., 2023). Our findings align with these reports as we also observed cued fear learning and memory deficits in Bmal1^+/−^ mice. Furthermore, we found that Bmal1^+/−^ mice exhibited decreased p-ERK/total ERK, p-MEK/total MEK, and p-CREB/CREB ratios, following cued fear memory training. These findings are consistent with previous studies linking impaired contextual fear memory to disruptions in MAPK phosphorylation (Eckel-Mahan et al., 2008). Hence, our results validate the functional role of Bmal1 in fear learning and memory and suggest that Bmal1 dysfunction may contribute to cognitive impairments in psychiatric and neurodegenerative disorders.

Bmal1 plays a crucial role in regulating neurotransmitter function and circadian rhythm, and its depletion disrupts circadian gene expression in brain nuclei, particularly those involved in neurotransmitter-related functions in the ventral striatum (Kanan et al., 2023; Zheng et al., 2023). It suggests that Bmal1 dysfunction may impact behaviors related to reward and motivation. Previous studies have demonstrated that Bmal1 deficiency can lead to neurodegeneration of dopaminergic neurons in the substantia nigra, a region crucial for dopamine production (Berbegal-Sáez et al., 2024). Additionally, Bmal1 deficiency disrupts gene expression in brain regions associated with reward and motivation, resulting in altered behavioral responses (Ali et al., 2019). Furthermore, Bmal1 knockdown affects astrocyte morphology, impairing synaptic coverage and neurotransmission (Ali et al., 2020), which may contribute to cognitive deficits. In addition, serotonin modulates circadian rhythms, with 5-HT1A and 5-HT1B receptors playing key roles (Meneses, 2014; 2017). However, the interplay between serotonin receptors, particularly 5-HT2CR, and Bmal1 remains unclear. Our study found significant alterations in 5-HT levels, which were rescued by SB242084, a selective 5-HT2C receptor antagonist, suggesting that inhibiting the 5-HT2C receptor may reverse the impairments in cued fear learning and memory observed in Bmal1^+/−^ mice.

Bmal1 plays a crucial role in regulating neuroinflammation across various neurological conditions (Liu W.-W. et al., 2020; Wang et al., 2020). Studies have demonstrated that Bmal1 deficiency exacerbates neuroinflammatory responses in MPTP-treated mice, leading to increased microglial activation and dopaminergic neuron loss (Liu W.-W. et al., 2020). Additionally, Bmal1 influences inflammatory gene expression in hypothalamic neurons (Tran et al., 2020). However, the interplay between 5-HT receptors, memory defects, and neuroinflammation is a critical area of research because neuroinflammation contributes to memory deficits and emotional disturbances, particularly in chronic pain conditions (Woodcock et al., 2020; Liu, 2022). In addition, studies have implicated serotonin receptors, including 5-HT2A, 5-HT6, and 5-HT7 receptors, in memory processes and alterations associated with neuropsychiatric disorders (Gasbarri et al., 2016). Similarly, dysfunctions in these receptors have also been linked to memory impairments in disorders like Alzheimer’s disease, and targeting 5-HT6 and 5-HT7 receptors with agonists or antagonists may be beneficial (Lorke et al., 2006). Our study provides further evidence for the link between Bmal1, neuroinflammation, and memory defects. We observed that Bmal1 haplodeficiency led to alterations in 5-HT2C receptor levels and neuroinflammatory responses. These findings suggest that targeting the 5-HT2C receptor may be a potential therapeutic strategy for mitigating cognitive impairments associated with Bmal1 dysfunction. To further investigate the role of inflammation in Bmal1-induced memory deficits, future studies could explore the effects of inflammation mediators like LPS on Bmal1 expression and function. In summary, Bmal1 haploinsufficiency leads to deficits in fear learning and memory, which are linked to alterations in neurotransmitters and receptors, particularly the 5-HT2C receptor. Targeting the 5-HT2C receptor may offer a potential therapeutic strategy for mitigating cognitive impairments associated with Bmal1 dysfunction. Further research is needed to explore the underlying mechanisms and develop targeted interventions.

## Data Availability

The original contributions presented in the study are included in the article/Supplementary Material; further inquiries can be directed to the corresponding authors.
