# Supplementary material for: Bmal1 haploinsufficiency impairs fear memory and modulates neuroinflammation via the 5-HT2C receptor
Source: Front Pharmacol. 2024 Nov 14;15:1422693. doi: 10.3389/fphar.2024.1422693 (PMC11602290; doi:10.3389/fphar.2024.1422693)
Supplement: Supplementary file 1 [file DataSheet1.DOCX]

**Figure S1. Production (A) and verification (B-C) of Bmal^+/-^ mice.**

**Figure S2. related to Figure 1. Bmal1 haploinsufficiency has no effect on spatial memory.** (A) The preference index in new object recognition (NOR) test. (B) Total distance moved in Y-maze test. (C) The alternation index (% of total triplet arm entries) in the Y-maze test. Bmal1^+/+^ mice, n = 7; Bmal1^+/-^ mice, n = 7. Data are reported as mean ± SEM. Unpaired t-test. ns, no significance.

**Figure S3. related to Figure 3. The effect of Bmal1 haploinsufficiency on the expression levels of protein synthesis-related proteins, synaptic plasticity-related proteins, mitochondrial proteins, and corticosterone in the PFC.** (A-C) Representative western blot images and protein levels of p-eEF2, p-eIF2α, p-eIF4E, PSD95, synaptophysin, SNAP25, p-TrkB, PGC-1α, TFAM, DRP1, OPA1, MFN1 in the PFC of Bmal1^+/+^ and Bmal1^+/-^ mice after cued fear conditioning paradigm. n = 4 - 6 mice. Data are reported as mean ± SEM. Unpaired t-test. ns, no significance; *p < 0.05; **p < 0.01. (D) The levels of corticosterone (CORT) in the PFC of Bmal1^+/+^ and Bmal1^+/-^ mice after cued fear conditioning paradigm. n = 6 - 7 mice. Data are reported as mean ± SEM. Unpaired t-test. ns, no significance; *p < 0.05; **p < 0.01.

**Fig. S4. related to Figure 4. The effect of VD or SB242084 treatment on the expression levels of mitochondrial proteins and p-AMPKα in the PFC of Bmal1^+/-^ mice.** (A-C) Representative western blot images and protein levels of complex I-V, p-AMPKα, PGC-1α, TFAM, DRP1, OPA1, MFN1 in the PFC of Bmal1^+/-^ mice after cued fear conditioning paradigm. n = 6 - 7 mice. Data are reported as mean ± SEM. Unpaired t test. Saline + Bmal^+/-^ group vs VD + Bmal^+/-^ group; Saline + Bmal^+/-^ group vs SB242084 + Bmal^+/-^ group. ns, no significance; *p < 0.05; **p < 0.01.
